
JOURNAL INFORMATION
==============================
NLM Title Abbreviation: ScientificWorldJournal
Journal ID: ScientificWorldJournal
NLM Journal ID: 101131163
Journal ID: TSWJ

The Scientific World Journal

ISSN: 2356-6140
EISSN: 1537-744X
Publisher: Wiley

ARTICLE INFORMATION
==============================
PMCID: PMC5823153
PMID: 19347229
DOI: 10.1100/tsw.2009.30
Article ID: 285979
Article version: 1
Subjects: Letter to the Editor

Thoracic Blastomycosis from Morocco

Lahmiti Saad *
Baki Salwa
University Hospital Mohammed VI Marrakech, Morocco
*Saad Lahmiti: lahmiti@gmail.comAcademic Editor: Klaus Kayser

Publication date: 2009
Electronic publication date: 2009 Mar 31
Volume: 9
First page: 200
Last page: 200
Received 2009 Feb 27; Accepted 2009 Mar 26
Copyright: Copyright © 2009 Saad Lahmiti and Salwa Baki.
Copyright year: 2009
License: This is an open access article distributed under the Creative Commons Attribution License, which permits unrestricted use, distribution, and reproduction in any medium, provided the original work is properly cited.
License URL: https://creativecommons.org/licenses/by/3.0/

==============================
Letter to the Editor
*TheScientificWorldJOURNAL* (2009) 9, 200
ISSN 1537-744X; DOI 10.1100/tsw.2009.30

TheScientificWorldJOURNAL
www.thescientificworld.com

### Thoracic Blastomycosis from Morocco

**E-mail**: lahmiti@gmail.com

*Received February 27, 2009; Accepted March 26, 2009; Published March 31, 2009*

---

We read with great interest the paper by Cheikh Rouhou et al., which was recently published online in *TheScientificWorldJOURNAL*[1]. In this article, the authors report a new case of pulmonary and vertebral blastomycosis from Tunisia. They found that the probable reservoir of *Blastomyces dermatitidis* is the soil. Blastomycosis is considered a rare and sporadic disease in Africa, unlike in some endemic regions in Canada or in the upper Midwest in the U.S.

In fact, few cases of the disease were reported in the African literature and only one case was reported in the Moroccan literature. In 2005, there was a case of thoracic blastomycosis. It was primarily suspected to be a pulmonary carcinoma, but was never histologically confirmed. All the biopsies performed showed inflamed material. The medical student taking care of the patient found out that his patient sells used clothes (he imports second-hand clothes from the U.S. and Canada, and resells them in his store). Therefore, our medical student suspected that the patient's symptoms could be due to an imported disease. The medical staff recommended performing a mycological study. Culture on Sabouraud's dextrose agar at 25°C further confirmed the presence of *B. dermatitidis northamericanus*. A mycological study was then performed on the clothes sold by the patient. It showed a considerable amount of blastomycosis spores, a finding that concludes that the infection was due to massive and chronic inhalation of the spores present in the imported clothes. The patient was treated with a daily 400-mg dose of fluconazole for 12 months, a treatment that led to a great clinical and radiological improvement. So far, the patient has shown no evidence of recurrence.

Our case illustrates two important facts: the first is that medical students play an important role in the diagnosis and treatment of new diseases in our university hospitals, and the second is that imported clothes are not always safe. There should be a strict and thorough verification of imported clothes in order to prevent deadly disease.

*Saad Lahmiti, M.D.*
*Salwa Baki, M.D.*
*University Hospital Mohammed VI Marrakech*

### REFERENCE

1. Cheikh Rouhou, S., Racil, H., Ismail, O., Trabelsi, S., Zarrouk M., Chaouch, N., Hantous, S., Khaled, S., El Mezni, F., and Chabbou, A. (2008) Pulmonary blastomycosis: a case from Africa. *TheScientificWorldJOURNAL* **8**, 1098–1103. DOI 10.1100/tsw.2008.141

---

**This article should be cited as follows:**

Lahmiti, S. and Baki, S. (2008) Thoracic blastomycosis from Morocco. *TheScientificWorldJOURNAL* **8**, 200. DOI 10.1100/tsw.2008.30.

---

*Corresponding author.
©2009 with author.
Published by TheScientificWorld; www.thescientificworld.com
